# Silica micro/nanospheres for theranostics: from bimodal MRI and fluorescent imaging probes to cancer therapy

**DOI:** 10.3762/bjnano.6.57

**Published:** 2015-02-24

**Authors:** Shanka Walia, Amitabha Acharya

**Affiliations:** 1 Biotechnology Division, CSIR - Institute of Himalayan Bioresource Technology (CSIR - IHBT), Post Box No. 6, Palampur (H.P.) 176 061, India

**Keywords:** bimodal imaging, fluorescence imaging, magnetic nanoparticles, organic dyes, quantum dots, silica nanospheres, theranostics

## Abstract

Nano-theranostics offer remarkable potential for future biomedical technology with simultaneous applications for diagnosis and therapy of disease sites. Through smart and careful chemical modifications of the nanoparticle surface, these can be converted to multifunctional tiny objects which in turn can be used as vehicle for delivering multimodal imaging agents and therapeutic material to specific target sites in vivo. In this sense, bimodal imaging probes that simultaneously enable magnetic resonance imaging and fluorescence imaging have gained tremendous attention because disease sites can be characterized quick and precisely through synergistic multimodal imaging. But such hybrid nanocomposite materials have limitations such as low chemical stability (magnetic component) and harsh cytotoxic effects (fluorescent component) and, hence, require a biocompatible protecting agent. Silica micro/nanospheres have shown promise as protecting agent due to the high stability and low toxicity. This review will cover a full description of MRI-active and fluorescent multifunctional silica micro/nanospheres including the design of the probe, different characterization methods and their application in imaging and treatment in cancer.

## Review

### 1 Introduction

In the modern era of medical diagnosis, X-rays have long played a major role in the clinical imaging of anatomical details of disease sites [1]. However, the development of suitable molecular diagnostic systems for cellular and sub-cellular imaging has remained a dream in medicine. In this respect, nanoparticles (NP) continue to receive attention in the field of medical imaging for their potential as specific contrast agents in vitro and in vivo. Clinical imaging can detect the disease sites at their very initial stages of growth and, thus, plays an integral part in medical diagnosis. All the currently available diagnostic imaging methods have their intrinsic advantages and disadvantages. The combination of multimodal imaging and theranostics will lead to cutting-edge technologies in which the potential of the NPs can be maximized. In our earlier review, we have compiled the literature reports on the biological studies of the hybrid nanocomposite materials, exclusively composed of luminescent quantum dots (QDs) and magnetically active iron oxide as bimodal imaging agents [2]. In this sense, nanostructured multimodal imaging probes for magnetic resonance imaging (MRI) and optical imaging are the most popular and interesting, since they provide high spatial resolution (MRI) and allow for a rapid screening of the disease site (optical imaging) simultaneously. But such hybrid nanocomposites have certain limitations, i.e., the magnetic NPs exhibit a very low chemical stability whereas the fluorescent part is quite photosensitive and has harsh cytotoxic effects. The problem can be solved by using silica micro/nanospheres as a protecting agent, which is chemically stable, less toxic and capable to reduce corrosion of fluorescent NPs and particle–particle magnetic bipolar interactions [3]. Further because of the large surface area, pore volume and easy functionalization, silica spheres have widespread applications as catalysts [4], in cosmetics [5], as nanocontainers [6], as nanoreactors [7], as adsorbing materials [8], for controlled delivery [9], and in biotechnology for the controlled release of biomolecules such as small drugs [10], therapeutic proteins [11], antibiotics [12], and antibodies [13].

In MRI, the relative difference of the signal intensity between two adjoining tissues can be improved by using different contrast agents (CAs). The ideal CA should be stable, tissue specific, less toxic with longer shelf life and a reasonable clearing period. The most common MRI CAs are paramagnetic chelated lanthanide complexes (positive contrast, *T*_1_-enhanced) and superparamagnetic iron oxide NPs (negative contrast, modified *T*_2_). Similarly the fluorescent CAs includes different lanthanide complexes, rare earth oxides, organic dyes, QDs, and ruthenium complexes. In this review article, we summarize recent literature reports on multifunctional nanocomposites for MRI and fluorescence imaging that are encapsulated with silica micro/nanospheres. Emphasis is put on synthesis, characterization and their simultaneous application in biomedical imaging and the diagnosis and therapy of cancer.

### 2 Different combinations of materials used for bimodal imaging

In order to optimize the use of silica micro/nanospheres as both fluorescent and MRI active probe, a broad range of nanostructures was investigated. The fluorescent and MRI responsive nanomaterials can be obtained through heterogeneous synthesis, and finally these can be encapsulated within silica spheres. The surface of the silica spheres can be further chemically modified with different targeting agents for a specific delivery of these nanocomposites.

#### 2.1 Lanthanide complexes as both magnetic and fluorescent probe

The electronic and magnetic properties of the lanthanide complexes are governed by 4f electrons. In some of the lanthanide complexes, the electronic transitions involved are of the type 4f*^n^*→4f*^n^*^−1^5d^1^, which accounts for their strong luminescence spectra. Further the high magnetic moment of the lanthanide complexes can be attributed to the spin–orbit coupling of the 4f electrons. Hence, the lanthanides complexes are fluorescent as well as magnetically active and, thus, were used extensively for designing multimodal imaging probes. Wu et al. [14] reported a simple reverse microemulsion method and coating process to synthesize silica-coated Gd_2_(CO_3_)_3_:Tb NPs. The synthesis was accomplished by using GdCl_3_ as a source of the Gd-complex and cetyl trimethylammonium bromide (CTAB), butanol and hexane as surfactant, co-surfactant and oil phase, respectively. The silica coating of the Gd_2_(CO_3_)_3_:Tb complex was done by using tetraethylorthosilicate (TEOS) under ammonical conditions. The synthesized NPs were characterized by HRTEM, EDX and FTIR. The size of the Gd_2_(CO_3_)_3_:Tb complex was found to be 8–12 nm with high degree of narrow size distribution. The coating with silica was confirmed by HRTEM studies in which the outer material covering the hybrid nanocomposites appeared as a thin layer with diameter of 6 nm. The magnetic properties of the NPs were measured by using a super conducting quantum interface device (SQUID) magnetometer. The photoluminescence spectra of silica coated Gd_2_(CO_3_)_3_:Tb NPs showed a characteristic charge-transfer band at 225 nm and a narrow peak at 274 nm. The *T*_1_-weighted MR images were obtained by using a 0.5 T magnet. The biocompatibility of these NPs was investigated by standard MTT cell proliferation assay. Studies suggested that the cell viability was maintained at 83% even after a high dose of 500 µg·mL^−1^ of the nanocomposites. To check the applicability of these nanocomposites as fluorescence imaging agents, Gastric SGC7901 cancer cells were treated and monitored under a fluorescence microscope at 405 nm excitation. Intense green fluorescence was observed from cancer cells which confirmed the internalization of NPs. Similarly, Pinho et al. [15] reported the synthesis of a bimodal MRI probe by embedding two lanthanide metal ions Gd^3+^ and Eu^3+^/Tb^3+^ inside silica NPs through Stöber’s process. In addition, the silica spheres were modified with a pyridine–based aromatic linkage which in turn was found to increase the quantum yield emission of Eu^3+^/Tb^3+^ ions present inside the silica shell. The silica NPs grafted with 3-aminopropyltriethoxysilane (APS) were activated with pyridine bismethylenenitrilotetrakis(acetic acid) (PMN) and, finally, to this different lanthanide salts were added. The characterization of these hybrid nanocomposites was done by TEM, fluorescence spectroscopy, ^13^C cross-polarization magic-angle spinning (CP/MAS) NMR and FTIR. The TEM micrographs suggested a uniform distribution of spherical nanocomposites with an average size of 67 ± 6 nm. Fluorescence spectra of solid SiO_2_@APS/PMN:Eu NPs showed an emission peak at 614 nm when excited at 270 nm. The prepared hybrid nanocomposite material was found to be rapidly internalized by RAW 264.7 cells. An increase in the intensity of *T*_1_-weighted MRI images of cellular pellets was observed when these nanocomposites were treated with the cells. The internalization into the cells was also monitored by fluorescence microscopy at 393 nm excitation.

**Figure 1:**
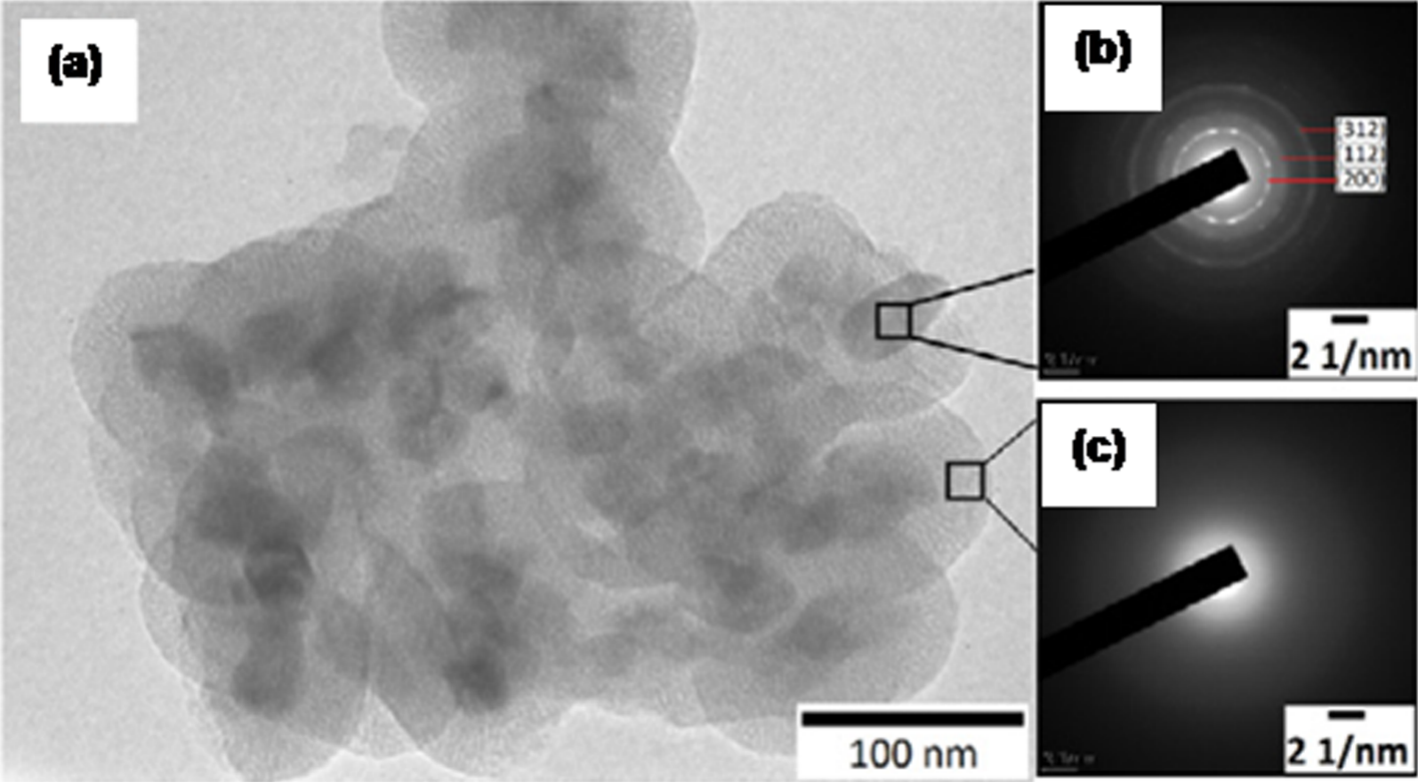
(a) YVO_4_:Eu^3+^ NPs encapsulated with mesoporous silica NPs. The insets show SAED patterns of (b) YVO_4_:Eu^3+^ and (c) mesoporous silica. Reproduced with permission from [16]. Copyright 2013 IOP science.

Recently, there has been a tremendous attention among the researchers in the field of rare-earth-doped NPs for multicolor phosphor applications. The extraordinary enhancement in the luminescent property (ca. 5–6 times of the initial value) of these nanoscale materials was associated with the hybridization of the electronic structure of dopant ions in a nanoparticle environment. Thus these new classes of materials can be used as potential fluorescent probes for biomedical imaging. Singh et al. [16] reported the synthesis of luminescent YVO_4_:Eu^3+^ NPs incorporated inside mesoporous silica NPs through a sol–gel process. The synthesis was enhanced by using Y(NO_3_)_3_·6H_2_O and Eu_2_O_3_ as reactants, CTAB as surfactant and TEOS as alkyl silicate. Finally, the anticancer drug doxyrubicin (DOX) was loaded into the prepared NPs. Characterization of mesoporous silica NPs (MSN), YVO_4_:Eu^3+^ NPs and YVO_4_-MSNs were done by XRD, FTIR, TEM and fluorescence spectroscopy. In case of MSN, a broad band was observed at 2θ = 22.0° which confirmed the presence of SiO_2_. The powder XRD pattern confirmed the tetragonal structure of YVO_4_ NPs. Similar peaks were also observed in case of YVO_4_-MSN nanostructures which inevitably lead to the conclusion that YVO_4_ retains its tetragonal structure even after silica encapsulation. Further FTIR studies were carried out to characterize the NPs. The absorption peaks at 800, 965, 1110 and 830 cm^−1^ were attributed to Si–OH bending vibrations, Si–OH, Si–O–Si stretching vibrations and V–O vibrations of YVO_4_, respectively. The size of MSN and YVO_4_:Eu^3+^ MSN NPs was studied by TEM and was found to be about 50 nm in both the cases. The amorphous nature of the silica surface and the crystalline nature of YVO_4_ NPs inside the silica spheres were further confirmed by selected area electron diffraction (SAED) (Figure 1). Mesoporosity studies of MSN and YVO_4_^_^MSN were done by thermogravimetric analysis. Fluorescence spectra of YVO_4_^_^MSN NPs showed two bands at 250 and 280 nm. The emission spectra of YVO_4_ exhibited a prominent peak at 618 nm. Drug loading efficiency of YVO_4_-MSN was examined with fluorescence intensity of DOX at 490 nm excitation. The loading efficiency was found to increase with increasing YVO_4_-MSN concentration. The drug release pattern also showed sustained drug release from the silica spheres. The cytotoxicity studies of DOX-loaded YVO_4_-MSN NPs were examined on two human cancerous cells, namely HeLa and MCF-7. Studies suggested that the cytotoxicity effect on MCF-7 was higher as compared to HeLa cells. Moreover, to induce the magnetic behavior, arginine-coated iron oxide NPs were mixed with DOX-loaded YVO_4_-MSN NPs. This biphasic mixture was studied for hyperthermia treatment in which the whole process was done at about 42 °C temperature under an applied AC magnetic field (250 kHz, 376 Oe). The results suggested that tumour cell death was low for DOX-loaded YVO_4_-MSN as compare to superparamagnetic ion oxide nanoparticles (SPIONs)-loaded DOX@YVO_4_-MSN in an oscillating AC magnetic field.

#### 2.2 Lanthanide complex as magnetic and organic dyes as fluorescent probe

**Figure 2:**
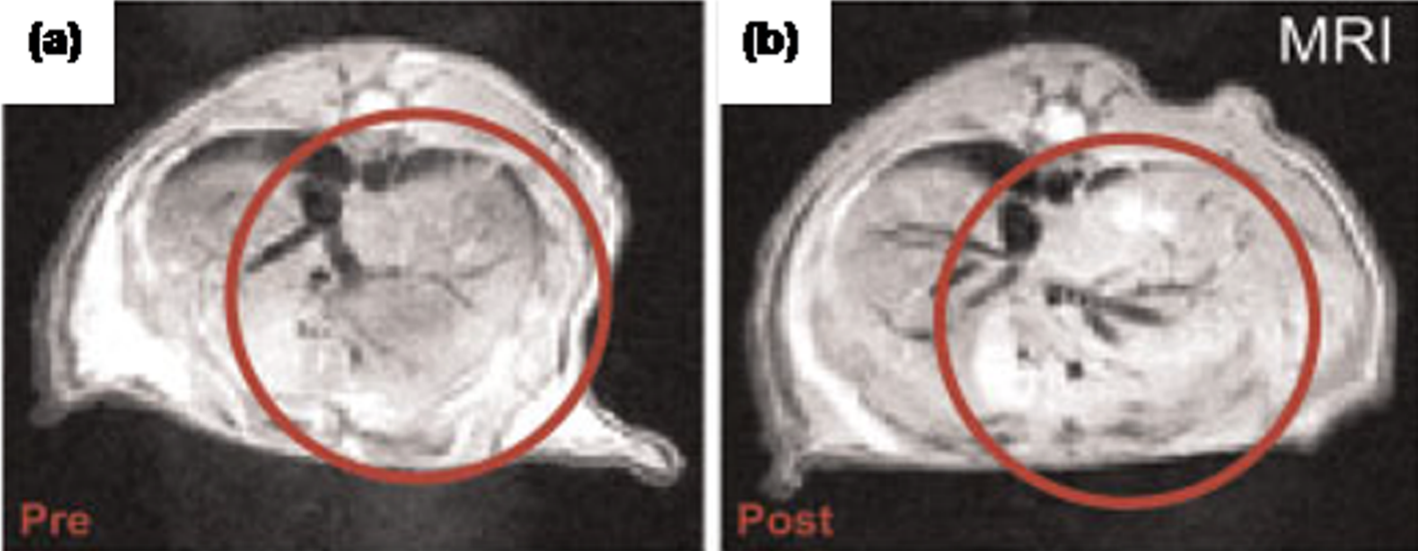
MR image of mice liver (a) prior to and (b) 24 h after injection of the trimodal NPs. Reproduced with permission from [18]. Copyright 2010 Wiley-VCH.

Organic dyes are the most common fluorophores and have been studied for long time. The most common fluorophores include fluorescein isothiocyanate (FITC) and rhodamine B (RhB). Kačenka et al. [17] reported the synthesis of hybrid NPs composed of La_0.75_Sr_0.25_MnO_3_ (LSMO) perovskite magnetite NPs encapsulated in a silica layer and to these NPs, a fluorescent FITC–APS (F) complex was covalently attached. These LSMO@SiF NPs were again coated with silica either by direct treatment with TEOS to form LSMO@SiF@Si-u or by reacting the centrifuged NPs with TEOS to form LSMO@SiF@Si-w. The secondary silica coating was expected to increase the stability of synthesized NPs. The excitation and the emission maxima of LSMO@SiF@Si-u were found to be at 492 and 514 nm, respectively. Further studies suggested that the excitation peak of LSMO@SiF@Si-u was more prominent than that of LSMO@SiF@Si-w, which was attributed to the fact that the latter contains less fluorescein. To check the biocompatibility of these nanocomposites, in vitro studies were carried out on HeLa cells and primary skin fibroblasts. The studies suggested that the HeLa cells showed higher viability (ca. 90%) compared to the fibroblasts cells (ca. 80%). Further, in case of the pancreatic islets (PIs) the cell viability was found to be more than 87%. Similarly, van Schooneveld et al. [18] reported a procedure for the synthesis of a trimodal contrast agent composed of gold/silica nanoparticle, organic dye (Cy5.5) and Gd-DTPA (gadolinium diethylene triamine pentaacetic acid) complex for simultaneous MRI, computed tomography (CT) and fluorescence imaging in vitro and in vivo (Figure 2).

#### 2.3 Lanthanide complex as magnetic and QDs as fluorescent probe

Among the inorganic fluorophores presently available, QDs have received much interest because of their ability to resist photobleaching. These nanocrystals were found to be brighter, more stable and to exhibit a narrower spectral distribution than organic dyes. Gerion et al. [19] reported a procedure for the synthesis of silica-shell-encapsulated hybrid nanomaterials consisting of paramagnetic Gd^3+^ ions and QDs or Au nanocrystals. The citric-acid-capped gold colloids and CdSe/ZnS QDs were silanized by using mercaptopropyltrimethoxysilane (MPS) as surfactant. Further, a Gd^3+^-DOTA (tetraazacyclododecanetetraacetic acid) complex was incorporated inside the SiO_2_ spheres. The concentration of the NPs and the number of the Gd^3+^ ions present in the silanized NPs were determined by absorption spectroscopy and inductively coupled plasma mass spectrometry (ICP-MS). The size of the NPs was found to be 8–15 nm. The MRI experiments suggested that the particle relaxivity was in direct correlation with the number of Gd^3+^ ions covalently linked to the silica shell. The in vivo studies further revealed that these silica-coated MRI probes enhance the MRI signal intensity in the bladder with no observable effects on the health of the animal. Similarly, Koole et al. [20] reported a procedure for the preparation of highly monodispersed silica particles with single core–shell–shell CdSe QDs incorporated into the center and paramagnetic Gd-DTPA-DSA [Gd-DTPA-bis(stearylamide)] in the lipid coating. The NPs were characterized by TEM, DLS and fluorescence studies. The size of the particles was found to be 31 ± 4 nm with an emission peak at approx. 620 nm. The in vitro studies with human umbilical vein endothelial cells (HUVEC) suggested that these NPs could be used simultaneously as fluorescent and MRI contrasting agent.

#### 2.4 Manganese oxide as magnetic and organic dyes/iridium complex as fluorescent probe

Due to their extremely low longitudinal relaxivity, manganese oxides, though less cytotoxic than Gd-complexes, have only a limited application potential as MRI CAs. Yang et al. [21] reported a chemical process for the preparation of magnetically active silica-encapsulated manganese oxide nanoparticles. In addition, rhodamine B isothiocyanate (RBITC) and folic acid (FA) were conjugated on the NP surface. These NPs showed absorption peaks at ca. 570 and 280 nm, which correspond to RBITC and FA, respectively. The MRI studies suggested that the synthesized nanocomposites exhibited a signal enhancement in the *T*_1_-weighted MRI images with increasing Mn concentration. The in vitro studies performed on HeLa cells suggested cell viability of more than 80% even at a Mn concentration of 50 mg·mL^−1^. The combination of results obtained from flow cytometry, confocal microscopy and MRI studies suggested that the prepared nanocomposites can be used for targeting cancer cells that overexpress folic acid. Similar strategies were also used by Peng et al. [22] by using an iridium(III) complex as fluorescent agent. Hu et al. [23] reported the synthesis of silica-encapsulated hydrophobic Mn_3_O_4_ NPs in which the silica surface was further modified by fluorescent rhodamine B and aptamer (AS411) as a targeting ligand. The in vitro confocal imaging and in vivo MRI studies showed that NPs specifically targeted the cancer cells. The histopathological and biochemical assays also confirmed low toxicity of Mn_3_O_4_@SiO_2_(RB)–PEG–Apt NPs. Again, Schladt et al. [24] designed PEG-functionalized silica-shell-coated manganese oxide NPs. Further the surface of these NPs was modified by using fluorescent APS–Atto 465. These nanocomposites were characterized by UV–vis and fluorescence spectroscopy, XRD, FTIR and SQUID magnetometry. The in vitro studies on bone-marrow-derived polymorphonuclear neutrophils (BM-PMNs) suggested that these nanoparticles exert toxic effects only at high concentrations. In a similar way, Chen et al. [25] synthesized FITC-conjugated mesoporous doxorubicin-loaded silica-encapsulated manganese oxide NPs. The TEM images showed that elemental Mn was equally distributed in the whole silica matrix. The in vitro and in vivo studies suggested that these nanocomposites can be used as a vehicle for pH-responsive intracellular release of doxorubicin.

#### 2.5 Iron oxide or lanthanide complexes as magnetic and ruthenium or lanthanide complexes as fluorescent probe

**Figure 3:**
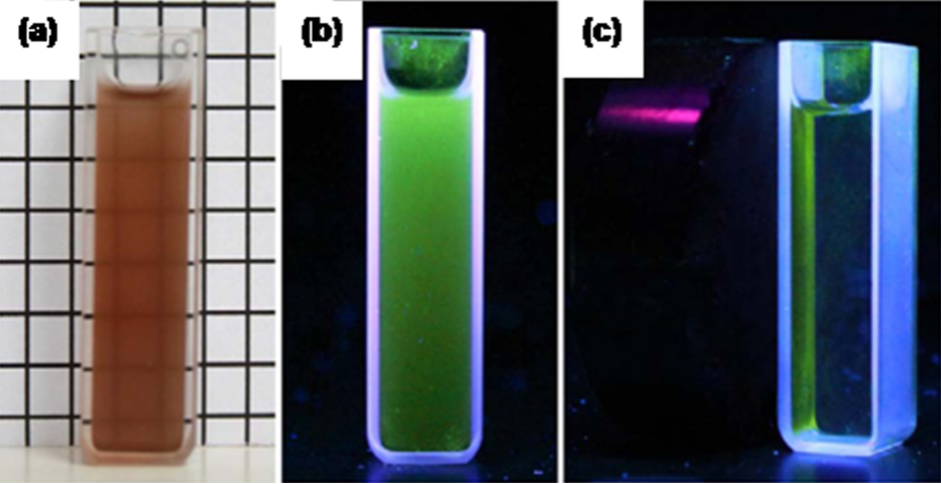
Magnetic and luminescent properties of core–shell-type hybrid NPs, Fe_3_O_4_@CeF_3_:10%Tb^3+^/SiO_2_, (a,b) before and (c) after magnetic capture, and (a) before and (b,c) during UV irradiation (λ = 254 nm). Reproduced with permission from [26]. Copyright 2011 Elsevier.

Superparamagnetic iron oxide NPs have been widely studied as MRI contrast agents for biological systems. These are less toxic compared to their chelated lanthanide counterparts. Again, because of the unique photoluminiscent properties, Ru(bpy) complexes were reported to be successfully used for different chemo-sensing applications. Runowski et al. [26] reported a new class of multifunctional fluorescent and magnetic-core-based nanocomposites synthesized through facile co-precipitation and microemulsion methods. Cerium–fluoride-doped terbium(III) NPs represented the luminescent part and Fe_3_O_4_ NPs were used as magnetic core. Both these nanostructures were trapped inside a silica shell, which acted as inert oxide. The synthesis involved simultaneous addition of luminescent NPs (CeF_3_:Tb^3+^) and magnetic materials (Fe_3_O_4_ NPs) inside silica shell in the presence of TEOS (Figure 3). The prepared NPs were characterized by XRD, TEM and fluorescence spectroscopy. The TEM images confirmed homogenous distribution of NPs of sizes 30–50 nm. When excited at 254 nm, the hybrid rare earth nanocomposites emitted green luminescent light due to Tb^3+^. In the excitation spectra, a peak centered at 254 nm was observed which was attributed to Ce^3+^→Tb^3+^ energy transfer transition.

**Figure 4:**
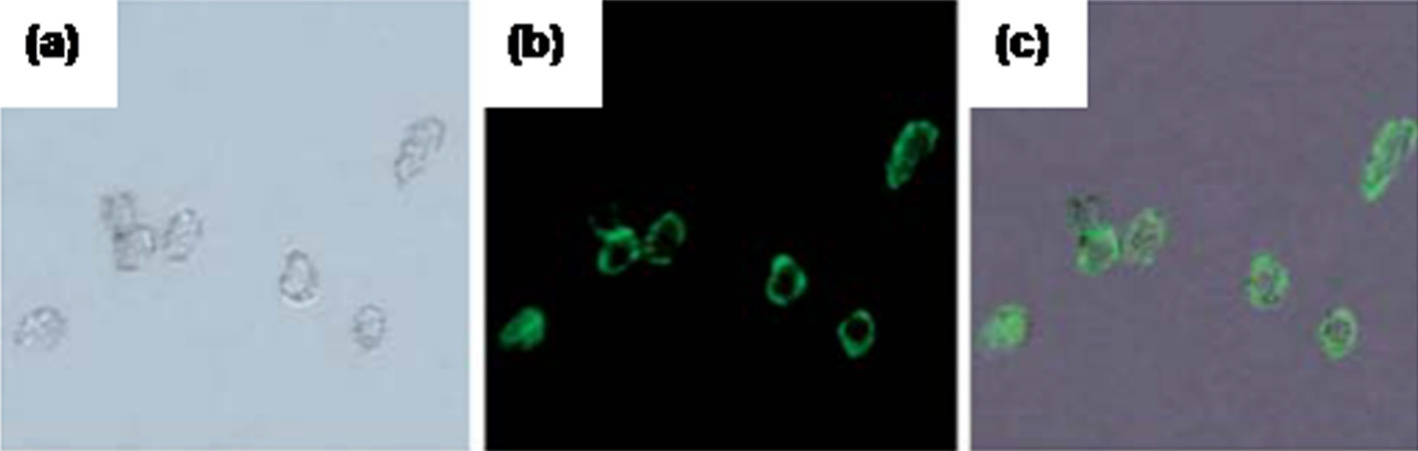
Images of live HeLa cells after being incubated with Fe_3_O_4_/NaYF_4_ nanocomposites biolabeled with transferrin. (a) Bright field, (b) fluorescent images in dark field and (c) superposition of (a) and (b). Reproduced with permission from [31]. Copyright 2010 Royal Society of Chemistry.

Zhang et al. [27] reported the synthesis of a hybrid nanocomposite material of magnetic and luminescent NPs inside silica spheres. The magnetic NPs (Fe_3_O_4_) were prepared by Massart’s method and finally these were coated with a silica layer by using TEOS. Further these Fe_3_O_4_@SiO_2_ NPs were encapsulated inside silica-coated luminescent Ru(bpy)_3_^2+^ shells (Ru(bpy)_3_ = tris(2,2'-bipyridyl)ruthenium(II) dichloride hexahydrate). The TEM images confirmed the formation of a nanocomposite material. The bifunctional NPs showed a slight red shift in the emission peak when compared with the Ru(bpy)_3_^2+^ complex. This was attributed to the possible interaction of silica with the Ru(bpy)_3_^2+^ complex. The magnetic studies suggested a typical superparamagnetic behavior for both iron oxide and the bifunctional nanocomposite with saturation magnetization values of 68.8 and 9.7 emu/g, respectively. To study the application of these NPs in an electrochemiluminescence sensor, these NPs were fabricated on a glassy carbon wafer in the presence of an external magnetic field. It was observed that the bifunctional NPs were distributed uniformly in the solution as long as there was an external magnetic field. The detection limit of tripropylamine (TPA) with these sensors was found to be 6.5 nM. Similarly, Santra et al. [28] reported the synthesis of multifunctional single-core/multiple-shell Ru(bpy):Gd(III)/SiO_2_ NPs. These nanocomposites were found to be highly photostable, radio-opaque and paramagnetic which allowed their use as multipurpose imaging probe. In a similar way, Viswanathan [29] reported a procedure for the preparation of hybrid nanocomposite material composed of silica coated iron oxide NPs and Ru(bpy) complex for multimodal imaging. Such strategies were also followed by Hu et al. [30], Mi et al. [31] (Figure 4).

Wu et al. [32] reported the synthesis of a hybrid nanocomposite material composed of Ru(bpy)-doped silica core–shell NPs and Pas (organic chromphore)–DTPA (chelate), through an water-in-oil microemulsion method. The average diameter of the prepared NPs was found to be 71 ± 4 nm with a silica core of 49 ± 3 nm as observed by TEM studies. The emission spectra of the nanocomposites displayed two peaks with Pas–DTPA at 545 and Ru(bpy) at 585 nm. Further, the Gd^3+^ and Tb^3+^ ions were also inserted in the silica matrix through a facile post functionalization. The presence of Gd^3+^ and Tb^3+^ inside silica shell was confirmed by inductively coupled plasma mass spectroscopy (ICP-MS). Likewise, Lu et al. [33] reported the synthesis of silica-coated hybrid NPs that can be used for different biomedical applications. The typical Stöber process was used for the fabrication of these nanocomposites. Initially, the ferrofluid magnetic NPs were prepared through a co-precipitation method and were coated with fluorescent NPs composed of chloride salts of rare earth and lanthanide metals, namely yttrium (Y), ytterbium (Yb) and erbium (Er) in presence of EDTA solution. The silica coating of these NPs was done by hydrolysis of TEOS and APS followed by conjugation of streptavidin on the silica surface. The TEM micrographs revealed that the particle size of the silica-coated NPs was 80–150 nm with uniform spherical shape. The magnetic characteristics of the synthesized NPs were studied by using a vibrating sample magnetometer (VSM). The saturation magnetization values of silica-coated and silica-free NPs were found to be 5.3 emu/g and 8.4 emu/g, respectively, whereas that of ferrofluid magnetic NPs was found to be 65 emu/g. The immobilization of streptavidin on the silica surface was confirmed by protein assay study on biotinylated goat anti-human IgG. Also, Choi et al. [34] reported the synthesis of magnetic and fluorescent inorganic NPs for biomedical applications. The synthesis was achieved by using iron oxide NPs, SiO_2_, lanthanide (Ln) salts (Eu, Tb) and 2,2'-bipyridine-4,4'-dicarboxylic acid (BDA), which acts as organic linker for binding Ln ions to the silica surface containing magnetic NPs. The average diameter of the fabricated NPs was 150 ± 10 nm as observed by TEM studies. The cubic spinel structure of Fe_3_O_4_ NPs was confirmed by XRD. The presence of the BDA linker was proved by observing SiO_2_/Fe_3_O_4_/BDA/Ln^3+^ complex by Raman spectroscopy which showed a prominent peak at 1306 cm^−1^ due to the stretching of carboxylate group in BDA after silica binding. When the PL spectra of SiO_2_/Fe_3_O_4_/BDA/Ln^3+^ and free Ln^3+^ ions were compared, the former showed higher luminescent intensity than the latter. Further, the magnetization of NPs was measured by SQUID studies and confirmed the superparamagnetic behavior of these hybrid NPs. In a similar way, Yan and Shao [35] reported a sol–gel process for the synthesis of lanthanide-activated magnetic silica nanostructures conjugated with polymer-modified zinc oxide NPs. The 2-hydroxyethyl-methacrylate-modified ZnO NPs and oleic-acid-functionalized iron oxide NPs were incorporated inside the silica spheres by using CTAB as template surfactant. Finally complexes of lanthanides, namely Eu^3+^, Tb^3+^ with nicotinic and isonicotinic acid were introduce inside silica. The characterization of these multifunctional nanostructures was achieved by UV–vis absorption spectroscopy, XRD, fluorescent spectroscopy, TEM, SEM, FTIR and VSM. In FTIR, the bands at 1633 and 1565 cm^−1^ were observed for both magnetic mesoporous silica ZnO NPs and its corresponding Eu complex, which confirmed their association with silica spheres. The size of ZnO-containing magnetic mesoporous silica nanospheres was about 200 nm as obtained from SEM and TEM images. Further the TEM images revealed that the core diameter of iron oxide NPs was about 10 nm. The luminescent spectra of Eu^3+^ hybrids was found to show a broad emission band between 200 and 500 nm with an emission maximum at 614 nm. Such strategies were also followed by Chen et al. [36].

#### 2.6 Iron oxide as magnetic and QDs as fluorescent probe

He et al. [37] reported a reverse microemulsion method for the synthesis of core–shell fluorescent magnetic silica-coated NPs. The Fe_3_O_4_ NPs were prepared by using FeCl_3_ and FeSO_4_ salts following co-precipitation method. The thioglycolic acid (TGA)-coated CdTe QDs were synthesized from CdCl_2_·5H_2_O. Finally, the desired nanocomposites were coated with silica in a reverse microemulsion process. To check the biological compatibility of these prepared hybrid nanocomposites, these were covalently conjugated with goat antimouse IgG (GM IgG). The characterization of the prepared nanocomposites was performed through TEM, UV–vis and photoluminescence (PL) spectrometry and fluorescence microscopy studies. The absorption peak for CdTe QDs was observed at 570 nm and was shifted to 540 nm after silica coating of NPs. This shift was also seen in the fluorescence spectra, in which the emission spectra shifted from 610 to 580 nm. Such shift in the absorption and emission spectra was attributed to a possible corrosion of CdTe QDs after silica coating. Magnetic properties of these hybrid nanocomposites were monitored at room temperature. The magnetization curve proved that the magnetic NPs retained their superparamagnetic property in the silica sphere, attaining a saturation magnetization value of 3.21 emu·g^−1^. It was found that after amine functionalization, the corresponding zeta potential peaks shifted from negative to positive values. Further studies suggested that the fluorescent intensity increases linearly with the concentration of hybrid nanocomposites-GM IgG concentration. Similarly, Sun et al. [38] reported a new class of magnetic fluorescent NPs in which CdTe NPs were conjugated covalently to superparamagnetic Fe_3_O_4_ NPs that were incorporated in silica and functionalized with a carboxylic acid group for bioconjugation. The synthesis involved coating silica around citric-acid-coated Fe_3_O_4_ NPs by using the Stöber process. The MPS-activated silica-coated Fe_3_O_4_ NPs were then conjugated with TGA-modified CdTe NPs. The silica-coated magnetic and fluorescent NPs were then conjugated with bovine serum albumin (BSA) by using *N*-hydroxysuccinimide (NHS) as cross linker. The synthesized NPs were then characterized by XRD, IR, TEM, absorption and fluorescence spectroscopy. The XRD diffraction peaks indicate the cubic structure for both Fe_3_O_4_ and CdTe NPs. A broad peak at 2θ = 20–25° proved the amorphous structure of silica. The IR spectral peak at 571 cm^−1^ confirmed the formation of Fe_3_O_4_ NPs. In TEM, the size of the Fe_3_O_4_/CdTe magnetic fluorescent nanocomposites was measured to be about 30 nm. The magnetic measurement was performed by using a vibrating sample magnetometer (VSM). The saturation magnetization of citric-acid-coated Fe_3_O_4_ NPs was found to be 40.97 emu/g, which was reduced to 9.90 emu/g after silica coating and was further decreased to 2.26 emu/g after addition of the CdTe QDs. For cell targeting studies, these nanocomposites were conjugated with anti-CEACAM8 antibody and were finally incubated with HeLa cells. Studies suggested that under UV radiation at 488 nm, the cells were successfully fluorescently labeled and imaged. Again, Jie-Mei et al. [39] reported the synthesis of bimodal Fe_3_O_4_@SiO_2_–CdTeS NPs for biomedical applications. Oleic-acid-stabilized Fe_3_O_4_ NPs were synthesized through a thermal decomposition method. CdTe QDs activated with mercaptopropionic acid (MPA), were prepared through a hydrothermal process. Further, the freshly prepared Fe_3_O_4_ NPs were coated with silica by using TEOS and finally amine functionalization was achieved by using APS. At last MPA-activated CdTe QDs were incorporated into a silica shell containing Fe_3_O_4_ NPs. The synthesized NPs were characterized by XRD, TEM, photoluminescence spectroscopy and magnetometry studies. In powder XRD, six diffraction peaks corresponding to the 220, 311, 400, 422, 440 and 511 planes confirmed the cubic geometry of Fe_3_O_4_ NPs. The silica coating on Fe_3_O_4_ NPs was confirmed by a strong peak at 2θ = 23°. The TEM images of Fe_3_O_4_/SiO_2_–CdTeS magnetic fluorescent NPs suggested that CdTeS QDs were anchored at the surface of mesoporous silica with several Fe_3_O_4_ NPs as cores. The saturation magnetization value of Fe_3_O_4_/SiO_2_ was found to be 13.57 emu/g and it was reduced to 9.09 emu/g for Fe_3_O_4_/SiO_2_–CdTe NPs. This suggested that the silica coating and the addition of CdTe QDs affect the magnetic properties of Fe_3_O_4_ NPs. Yet again, Wang et al. [40] reported a simple reverse microemulsion method for the synthesis of magnetic and fluorescent silica nanostructures. The synthesis involved the incorporation of magnetic iron oxide and mercaptosuccinic-acid-coated fluorescent CdTe NPs into a silica shell. The surface of the silica shell was then amine-functionalized through APS treatment for bioconjugation with FITC-IgG by using glutaraldehyde as linker. The characterization of the prepared NPs was carried out through XRD, UV–vis spectroscopy and PL emission spectra, VSM, TEM and SEM. The fluorescence spectra of mercaptosuccinic-acid-capped CdTe QDs exhibited an emission peak at 585 nm, which was blue shifted when either the QDs were inside the silica sphere or the concentration of the iron oxide was increased. Both the TEM and SEM micrographs confirmed a uniform size for the hybrid nanocomposite materials with a diameter of 50 nm. The fluorescence emission peak at 520 (from FITC) and 573 nm (from nanocomposites) confirmed the bioconjugation of amine-functionalized hybrid nanocomposites with FITC-IgG. In a similar way, Nai-Qiang et al. [41] reported a simple modified Stöber process in combination with layer by layer assembly for the preparation of nontoxic bimodal magnetic and luminescent nanocomposite materials consisting of Fe_3_O_4_ NPs and Mn-doped ZnS QDs. The synthesis was achieved by depositing a silica shell over Fe_3_O_4_ NPs with TEOS. Further, Mn-doped ZnS QDs were bonded with APS activated Fe_3_O_4_@SiO_2_ NPs through electrostatic interactions. The prepared NPs were characterized by X-ray analysis, X-ray photoelectron sepectroscopy (XPS), TEM, PL spectroscopy and vibrating sample magnetometer. In XRD, five diffraction peaks were observed that were assigned to 220, 311, 400, 511 and 440 planes. These results suggested the incorporation of magnetic NPs inside silica spheres. Similarly, the lattice plane spacings assigned to 111, 220 and 311 planes represented the cubic structure of ZnS QDs. The XPS data suggested the conjugation of Mn-ZnS NPs with SiO_2_/Fe_3_O_4_ NPs. The TEM micrographs showed average diameters of Fe_3_O_4_/SiO_2_ NPs of 100–130 nm. The fluorescence emission peak of Mn-ZnS QDs was observed at 460 nm. Further, a blue shift of this peak by 10 nm was observed when Mn-ZnS QDs were added to magnetic silica sphere. The magnetic properties of these NPs were observed by SQUID. The saturation magnetization value of Fe_3_O_4_ magnetic NPs doped inside silica was found to be 7.0 emu/g, which was far less than the saturation magnetization of the magnetite/maghemite NPs used for the preparation of these core–shell spheres (54 emu/g). This decrease in magnetic saturation was attributed to the presence of a thick silica shell in the former nanocomposites. Likewise, Ruhland et al. [42] reported the synthesis of size-controlled magnetic and fluorescent core–shell hybrid NPs coated with a protective silica sheath through a thermal decomposition process. The synthesis involved the incorporation of Fe_2_O_3_ and CdSe/ZnS NPs into silica that was then activated by silane-carrying methacrylate and 3-(methacryloyloxy)propyltrimethoxysilane (MPTS). Finally, a lightly crosslinked poly(*N*-isopropylacrylamide) (PNIPAAm) shell was immobilized by a graft polymerization process. The characterization of these NPs was carried out through DLS, TEM, VSM, and UV–vis and fluorescence spectroscopy studies. The TEM micrographs and EDX studies clearly indicate the encapsulation of both Fe_2_O_3_/CdSe(ZnS) NPs inside the silica shell. In cryo-TEM studies CdSe(ZnS)/SiO_2_/PNIPAAm NPs displayed a fuzzy corona-like structure. All these studies lead to the conclusion that the particles retain their characteristic properties even after their conjugation inside the MPTS-coated silica corona. Again, Selvan et al. [43] reported the synthesis of multifunctional silica-capped magnetic and fluorescent NPs that can be used for biolabelling, magnetic resonance imaging, single-electron transistors, lasers and light emitting diodes. The synthesis of magnetic QDs (MQDs) involved the simultaneous addition of CdO, stearic acid and Fe_2_O_3_ NPs in trioctylphosphine medium. These MQDs were then capped with silica by using APS (as stabilizer) and tetramethylammonium hydroxide (TMAH). Finally, bioconjugation on silica surface of the magnetic NPs and quantum dots was accomplished by using oleyl-*O*-poly(ethylene glycol)succinyl-*N*-hydroxysuccinimidyl ester. The characterization of these NPs was done by using UV–vis and fluorescence spectroscopy, HRTEM, STEM and SQUID studies. The absorption and emission spectra suggested that the QDs retained their fluorescent properties even after silica coating. The heterodimer structure of MQD was confirmed by HRTEM studies. Finally, the fluorescence properties of these composites were tested with three different cell lines, namely HepG2 human liver cancer cells, NIH-3T3 mouse fibroblast cells, and 4T1 mouse breast cancer cells. Confocal laser scanning microscopy (CLSM) images indicated a successful bioconjugation of silica-coated QDs and MQDs with a bio-anchored membrane. Again, Salgueiriño-Maceira et al. [44] reported a new class of multimodal magnetic and fluorescent silica spheres synthesized through the Stöber process and a layer-by-layer assembly approach. The incorporation of Fe_3_O_4_ NPs into the silica shell was done following the Stöber process by using TEOS/NH_4_OH. The layer-by-layer assembly approach was utilized for the deposition of CdTe QDs inside the silica shell. The TEM micrographs showed the average diameter of 220 ± 10 nm for these nanocomposites. There was a blue shift of approximately 5 nm in the emission spectra of CdTe QDs in the magnetic silica sphere compared to simple CdTe QDs. Similar changes were also observed in the CdTe PL spectra in which a shift of about 10 nm was monitored after silanization. The saturation magnetization value was found to be 1.34 emu/g for the hybrid nanocomposite material, which was relatively low compared to the simple magnetic NPs. This observation was in line with the fact that in the hybrid nanocomposite material the thick silica coating acts as diamagnetic component. Similarly, Yi et al. [45] reported the synthesis of silica-coated γ-Fe_2_O_3_ magnetic NPs and CdSe QDs through a reverse microemulsion approach. The synthesis involved the isolated preparation of magnetic NPs and QDs that were then encapsulated inside silica by using cyclohexane and TEOS. The TEM micrographs clearly indicated the incorporation of both types of NPs inside the silica shell. Such encapsulation studies were carried out at different time intervals but only after 48 h the spherical shaped silica nanospheres were obtained. In addition, the absorption and emission peaks for CdSe were obtained at 530 nm and 554 nm and were found to blue shift after silica encapsulation. The magnetic property of these nanocomposites was confirmed by SQUID studies. Ruan et al. [46] reported the synthesis of biocompatible hydrophilic silica-capped magnetic NPs and QDs through a reverse microemulsion method. The Fe_3_O_4_–polystrene NPs and CdTe QDs were conjugated and then incorporated into the silica core. The characterization and structure elucidation of the prepared NPs were done by using SEM, TEM, PL spectra and SQUID analysis. The sizes of the nanocomposites were found to be ca. 150 nm with about 30 nm silica shell thickness. The PL spectra of these hybrid nanocomposites were found to shift by about 5 nm when compared with free CdTe QDs. The SQUID studies suggested that the saturation magnetization value of the synthesized NPs was 4.0 emu/g. To observe the biocompatibility of these nanocomposites, both in vitro and in vivo studies were performed with HEK293 cells (human embryonic kidney 293 cells) and mice, respectively. Studies on HEK293 showed reasonable growth on treatment with 50 µg/mL dose of these hybrid nanocomposites which in turn confirmed the low cell toxicity level of the prepared nanocomposites. The biodistribution studies of such nanocomposites in mice showed that these were mainly located in lung, liver and spleen without any trace in the brain tissues. These results suggested that the prepared nanocomposites could not get through the blood brain barrier. The TEM and histopathological analysis of the liver tissues suggested these nanocomposites were mainly expelled out from the mice body possibly by liver secretion. In a similar way, Guo et al. [47] reported the synthesis of hybrid nanostructures of poly(*N*-isopropylacrylamide)-coated luminescent/magnetic silica microspheres. The synthesis involved a sequence of three steps in which the first step led to the incorporation of iron oxide magnetic NPs inside silica spheres through the Stöber process. In the next step, negatively charged TGA-functionalized CdTe QDs were conjugated with the magnetic NPs inside the silica shell. Finally, the silica-coated NPs were wrapped with thermosensitive poly(*N*-isopropylacrylamide) by a template polymerization process. The average diameter of silica-coated magnetic NPs was found to be 100 ± 10 nm as studied by TEM analysis. It was observed that after the addition of QDs, the average diameter of the particles increased by about 20 nm. The silica-coated QDs showed a red shift of 5 nm in the emission spectra when these were compared with free QDs. The confocal microscopy images of CHO cells treated with hybrid nanocomposites suggested the presence of the aggregates inside the cells. Kim et al. [48] reported the synthesis of magnetic and fluorescent NPs, encapsulated inside silica through a sol–gel process. The synthesis involved the encapsulation of oleic acid-capped magnetite NPs and CdSe/ZnS QDs, inside the silica spheres. Both FE-SEM and TEM images suggested that the average diameter of the magnetite–mesoporous silica spheres was around 150 nm. The mesoporous property of the prepared NPs was demonstrated by N_2_ adsorption/desorption isotherm studies and the pore size was found to be ca. 3.5 nm. The drug release profile of ibuprofen suggested that the drug release rate can be controlled by modifying the surface of the silica spheres. Further, Insin et al. [49] reported a sol–gel process for the synthesis of hybrid NPs embedded inside silica microspheres for fluorescence and magnetic resonance imaging. The maghemite (γ-Fe_2_O_3_) NPs were prepared by using 1,2-hydroxydodecanoic acid and then these were coupled with CdSe/ZnS QDs. Finally, silica coating was achieved by using TEOS. The TEM and SEM images confirmed the incorporation of NPs inside silica. The presence of QDs was confirmed by spectrophotometric analysis whereas the inductively coupled plasma optical emission spectroscopy was used to determine the amount of QDs and magnetic NPs inside the silica shell. The studies suggested 4600 ± 1400 QDs and 13000 ± 3700 magnetic NPs for each microsphere. Such strategies were also followed by Xiao et al. [50], Gong et al. [51], Law et al. [52], Xu et al. [53].

#### 2.7 Iron oxide NPs as magnetic and organic dyes as fluorescent probe

Zhang et al. [54] reported a conventional sol–gel method with a reverse microemulsion approach for the synthesis of luminescent magnetic silica nanotubes. The synthesis involved the deposition of a silica layer on aluminium oxide and then to this solution trimethoxy(octadecyl)silane (C18-silane) was added. Next, to this silica microemulsion mixture the fluorescent material rhodamine B (RhB) was added. Finally, Fe_3_O_4_ NPs were introduced inside gold-capped nanotubes. The structure and properties of synthesized nanotubes were studied by TEM and the results suggested that the thickness of fluorescent layer was 1–2 nm. It was also observed that the photostability of RhB was higher in the silica matrix compared to that in the nanotubes voids. The presence of magnetic NPs was reported to have decreased the fluorescence intensity of RhB. Similarly, Zhang et al. [55] reported a click-chemistry method to incorporate RhB into paramagnetic silica NPs. The magnetic iron oxide NPs were prepared by a co-precipitation method and finally these were covered by amino-modified silica spheres through the Stöber process using TEOS. Subsequently, the prepared nanocomposites were carboxy-modified with glutaric anhydride and then converted to Fe_3_O_4_@SiO_2_–C≡C NPs by using propargylamine. Finally, the click-chemistry reaction was performed on the NP surface by using RhB-N_3_ in the presence of copper sulfate. The characterization of the prepared NPs was performed through XRD, XPS, TEM, FTIR, VSM and fluorescence spectroscopy studies. The XRD studies confirmed the cubic crystal structure of the magnetic NPs. The broad peak around 2θ = 20° proved the amorphous structure of silica shell. The average particle size of magnetic NPs and Fe_3_O_4_@SiO_2_ NPs was found to be 9 and 13 nm from XRD which was similar to the sizes (12–14 nm) obtained by TEM micrographs. The FTIR peak at about 2108 cm^−1^ confirmed the coupling of RhB and the C≡C bond to the Fe_3_O_4_@SiO_2_ surface. The emission peak of fluorescent RhB at 640 nm was found to blue shift to 580 nm in the case of Fe_3_O_4_@SiO_2_ NPs. The saturation magnetization value of the magnetic nanoparticles was observed to be 21 emu/g. The biocompatibility of these NPs was determined by incubating them with the human fibroblasts cells for which a cell viability of about 90% was observed even after 24 h. Again, Liong et al. [56] reported the synthesis of multifunctional silica nanospheres with magnetic iron oxide NPs, luminescent fluorescein isothiocyanate (FITC) and camptothecin or paclitaxel as anti-cancer drug molecules entrapped inside the core. These nanospheres were finally modified with folic acid for targeting two different pancreatic cancer cell lines, namely PANC-1 and BxPC3. The confocal microscopy studies suggested that the nanocomposites were indeed internalized by the cells and not simply bound on the surface membrane. The *T*_2_-weighted images suggested that the NPs can be used as MR contrast agent in solution and in cells. Further, Chekina et al. [57] reported the synthesis of bifunctional NPs through a silinazation process. The maghemite (γ-Fe_2_O_3_) NPs were prepared by oxidizing pure magnetite NPs with sodium hypochlorite. The magnetic NPs were then coated with silica by hydrolyzing them with TEOS, followed by APTES and carboxymethyl chitosan (CMSC) coating. Finally, the nanocomposites were made fluorescent by FITC labeling. In a second approach, the γ-Fe_2_O_3_ NPs were modified through a CMSC coupling reaction. The characterization of the prepared hybrid nanocomposites material was done by attenuated total reflectance FTIR (ATR FTIR), TEM, DLS, fluorescence and atomic absorption spectroscopy (AAS) studies. The TEM micrographs suggested that the average diameter of CMSC coated γ-Fe_2_O_3_ NPs and bifunctional silica-coated NPs were 9 nm (PDI = 1.17) and 9.3 nm (PDI = 1.15), respectively. The iron content in the given samples measured by AAS was found to be 48% by weight in Fe_2_O_3_–CMCS NPs and 36% by weight in Fe_2_O_3_–SiO_2_-AP-CMSC NPs. The presence of the silica layer was proven by ATR FTIR spectra which exhibited a strong peak at 800 cm^−1^ related to the symmetric stretching vibration of Si–O–Si. The FITC conjugation with hybrid NPs was studied by using fluorescence spectroscopy. Both FITC and FITC-γ-Fe_2_O_3_ NPs exhibited an emission peak at 516 nm. The biocompatibility of these hybrid NPs was observed by incubating rat mesenchymal stem cells (rMSCs) with these hybrid NPs. The best results were found for FITC-labeled γ-Fe_2_O_3_–SiO_2_-AP-CMCS NPs which showed a cell labeling of about 64%. Further these hybrid nanocomposites were injected into a rat brain to evaluate their applicability for MR imaging in vivo. The results suggested that the cell implant was visible as hypointense spot with excellent contrast against the surrounding tissues. Again, Corsi et al. [58] reported the synthesis of a modified hybrid nanocomposite material consisting of magnetic fluorescent NPs for bimodal diagnosis of breast cancer cells. The magnetic Fe_3_O_4_ NPs were prepared by using a co-precipitation method and then coated with silica. Further these nanocomposites were treated with FITC-APTES and TEOS to obtain amine-functionalized silica-coated fluorescent magnetic NPs. The prepared NPs were then further functionalized with MeO-PEG-NH_2_ using glutaraldehyde as linker. The characterization of the NPs was performed through TEM, DLS and spectrophotometric analysis. At a concentration of 50 µg·mL^−1^, the PEG-conjugated nanocomposites did not show any cytotoxic effect towards MCF-7 cells even after 5 h of incubation. The TEM images showed localization of these particles in small subplasmalemmal vesicles without any appreciable amount of change in the NP size and shape. In a similar way, Chen et al. [59] reported the synthesis of multifunctional magnetic and fluorescent nanocolloids. The synthesis began with the preparation of either inorganic QDs or organic semiconductors. These nanocomposites were transferred to APTES-modified silica shells that were doped with magnetic FePt NPs. Further, tetramethyl rhodamine isothiocyanate (TRICT) was inserted in the outer SiO_2_ shell to prevent the risk of leakage of the dye from the magnetic core. The nanocolliod showed photoluminescence emission (PL) at ca. 575 and 625 nm. The corresponding absorption spectra showed a slight decrease in absorbance at 350 nm compared to free TRICT. The photoluminescence lifetime of TRICT attached magnetic NPs showed a temperature variation of ca. 20 ps/°C. This result suggested that water soluble hybrid nanocolloids can be used as probes for remote measurement of the temperature of aqueous media. Similarly, Wang et al. [60] reported the synthesis of a multifunctional hybrid nanostructures and they have shown their application in photodyanamic therapy. The co-encapsulation of magnetic NPs and fluorescent dye inside the silica shell was accomplished by a reverse micelle method. The successive reaction steps involved the coating of nonporous silica layer on iron oxide NPs and conjugating these with FITC. Then, these nanocomposites were again coated with mesoporous silica by using APTES and finally attached to folic acid. The average diameter of Fe_3_O_4_@SiO_2_ was found to be 40 ± 5 nm whereas the thickness of mesoporous silica was ca. 9 nm as studied by TEM. The Brunauer–Joyner–Halenda (BJH) pore-size distribution indicates that the hybrid nanocomposite material has uniform mesopores with an average pore size of 2.5 nm. When the hybrid nanocomposite material was excited at 488 nm, it exhibited only one emission peak at 520 nm corresponding to fluorescein. The effectiveness of these nanocomposites to generate singlet oxygen (^1^O_2_) upon light irradiation was studied using 1,3-diphenylisobenzofuran (DPBF) by UV–vis spectroscopy. The decrease in the absorbance band at 400 nm suggested the successful photogeneration of singlet oxygen. The biocompatibility of these nanocomposites was studied by incubating these nanocomposites with human hepatocyte cells (QSG-7701) for 24 h. The results indicated that even after 800 µg·mL^−1^ of concentration of the nanocomposite, the cell viability was more than 60%. Further after 14 h of incubation with the NPs, the cells were irradiated with a 660 nm laser (75 mW·cm^−2^) for 2.5 min and 10 min, respectively. It was found that almost all the cells died after 10 min which in turn proved the fact that these nanocomposites can be used for photodynamic therapy. Again, Lu et al. [61] reported the water-in-oil reverse micelle method for the fabrication of magnetic silica-coated fluorescent nanostructures. The synthesis was initiated by preparing the oleic-acid-coated iron oxide NPs by using the seed growth method. These NPs were then reacted with TEOS and *N*-1-(3-trimethoxysilylpropyl)-*N*-fluoresceylthiourea to form the shell structure attached to FITC. The TEM micrographs showed that the core–shell structure for the NPs with an average size of 50 nm with a 10 nm core. It was found that the NPs emitted green light at 510 nm. The magnetic studies suggested that the nanocomposites exhibited typical property of superparamagnetic iron oxide by shortening the relaxation time *T*_2_. The cell uptake experiment of these nanocomposites was performed with human mesenchymal stem cells (hMSCs) at different time and dose intervals. The results showed that NPs were accumulated fast by the cells, which can be clearly detected by MRI. The in vivo studies carried out on nude mice reflected that the nanocomposite labeled cells could be detected in living animals under a clinical MRI system. Further, the adverse effects of these nanocomposites on the functions of stem cells were examined on adipocytes and osteocytes. Such strategies were also followed by Teng et al. [62], Kim et al. [63], Lee et al. [64], Shen et al. [65]. Further, Nagao et al. [66] reported the synthesis of a hybrid nanocomposite material composed of a magnetic silica core and a fluorescent shell by combining two different techniques, namely heterocoagulation and soap-free emulsion polymerization. The *N*-trimethoxysilylpropyl-*N*,*N*,*N*-trimethylammonium chloride (TSA)-modified Fe_3_O_4_ NPs were prepared by the Massart method and finally coagulated with silica NPs prepared by using TEOS following the Stöbers method. Finally, the fluorescent polymer shell prepared by 3-methacryloxypropyltrimethoxysilane (MPTMS) was conjugated with the magnetic NPs. The characterization of these NPs was achieved by using TEM, TGA, VSM, powder XRD, fluorophotometer and DLS studies. The saturation magnetization value of magnetic NP@SiO_2_FITC core was found to be 6.7 emu/g.

## Conclusion

The non-specific uptake several of drugs by healthy tissues/organs was found to be the major cause of severe side effects in the body of a patient. Bio-coating of these drugs will reduce the side effects significantly, since the carrier can protect organs from toxic drugs and prevent the decomposition/denaturation of the drugs prior to reaching the targeted cells. Further this will also allow drugs to be released in a controlled way [67]. Silica micro/nanospheres have unique characteristics of high surface area, tunable pore size and volume and well-defined surface properties for bioconjugation. These properties allow such materials to be a more biocompatible coating agent than the regular organic molecules. The incorporation of magnetic and fluorescent nanocomposite material and therapeutic molecules inside the silica spheres will allow these NPs to be used for simultaneous multimodal imaging as well as for therapy.

Because of the low photo-stability, narrow absorption spectra and broad emission bands, the in vivo imaging applications of organic dyes are limited [68–69]. QDs, being brighter and more photoresistant than organic dyes, have gained tremendous attention for imaging of biological systems. But due to their cytotoxic effects they can cause damage to the healthy tissues. Similar toxicity results were also reported for rare earth oxides, Ru complexes and other fluorescent lanthanide complexes. Again the common CAs for MRI are toxic Gd complexes whereas the superparamagnetic iron oxides are less stable. Hence to develop a magnetic and fluorescent probe for multimodal imaging requires the appropriate selection of the magnetic and the fluorescent component, which will be chemically stable as well as less toxic and photoresistant.

In this review we focused on silica-coated hybrid magnetic fluorescent NPs and their applications in biomedical imaging. The different strategies applied for the synthesis of these multifunctional nanocomposites was discussed along with the in vitro and in vivo results. Currently, there is no commercialized product available in market for multimodal imaging. This is possibly because there are still many issues that need to be addressed. The chemical stability of these nanocomposites is one of the major drawbacks since the processing in biomedical applications requires long time durations. Further, the toxicity is another concern when advocating NPs for any clinical use. Unfortunately, the toxicity of nanomaterials is still very poorly studied and understood. The control of the NP size is also very important since higher sizes may limit the access to intracellular organelles and alter the intrinsic activity of any attached proteins. Hence, it is highly desirable to appropriately design the chemical processes for surface functionalization in order to maintain a small overall size of the NPs. In addition, the non-specificity associated with nanoparticle therapy is a big issue. With regard to this, the magnetic fluorescent NPs can be helpful because these can be dragged by using an external magnetic field to the targeted area and can be kept there until diagnosis or treatment are complete. Finally, they can be removed from the site. All these steps can be monitored by MRI and fluorescence microscopy simultaneously, which will allow for the full control over the processes. Thus, further development and utilization of magnetic fluorescent nanoprobes could revolutionize many aspects of modern medicine [70].
